# Quercetin: A Molecule of Great Biochemical and Clinical Value and Its Beneficial Effect on Diabetes and Cancer

**DOI:** 10.3390/diseases10030037

**Published:** 2022-06-29

**Authors:** Aikaterini-Spyridoula Michala, Agathi Pritsa

**Affiliations:** Department of Nutritional Sciences and Dietetics, School of Health Sciences, International Hellenic University (IHU), P.O. 141 Sindos, 57400 Thessaloniki, Greece; inami2102@gmail.com

**Keywords:** quercetin, flavonol, antioxidant, diabetes, cancer

## Abstract

Quercetin belongs to the broader category of polyphenols. It is found, in particular, among the flavonols, and along with kaempferol, myricetin and isorhamnetin, it is recognized as a foreign substance after ingestion in contrast to vitamins. Quercetin occurs mainly linked to sugars with the most common compounds being quercetin-3-O-glucoside or as an aglycone, especially in the plant population. The aim of this review is to present a recent bibliography on the mechanisms of quercetin absorption and metabolism, bioavailability, and antioxidant and the clinical effects in diabetes and cancer. The literature reports a positive effect of quercetin on oxidative stress, cancer, and the regulation of blood sugar levels. Moreover, research-administered drug dosages of up to 2000 mg per day showed mild to no symptoms of overdose. It should be noted that quercetin is no longer considered a carcinogenic substance. The daily intake of quercetin in the diet ranges 10 mg–500 mg, depending on the type of products consumed. This review highlights that quercetin is a valuable dietary antioxidant, although a specific daily recommended intake for this substance has not yet been determined and further studies are required to decide a beneficial concentration threshold.

## 1. Introduction

Dietary flavonoids such as quercetin, kaempferol, and apigenin are considered to have stronger antioxidant activity in comparison to popular antioxidants, e.g., vitamin C and E [1]. Furthermore, flavonols are phenolic compounds that belong to the subclass of flavonoids and are considered one of the most abundant phenolic compounds of the plant kingdom. Although the concentration and variety of flavanols in plants is quite high, they are largely absent from algae and fungi [2]. The main compounds in the class of flavonols are kaempferol, isorhamnetin, myricetin, and finally, quercetin. They are most commonly found in plants as O-glucosides, which are linked molecules of cyclic carbon with sugars. The sugar component is usually bound to the C-ring at various positions (3′, 4′, 5′, 7′) [3]. Flavonols, like all flavonoids, occur in nature in two forms, either as an aglycone lacking a carbohydrate moiety, or like a glycoside, where the hydroxyl group of the carbonate ring has been replaced by sugars such as glucose, rhamnose, or rutinose [4]. Quercetin has been reported to constitute 60–75% of total dietary flavonoid and flavonol intake [5,6]. According to the International Nomenclature System of the International Union of Pure and Applied Chemistry (IUPAC), the formal name for quercetin is 2-(3,4-dihydroxyphenyl)-5,7-dihydroxy-4H-1-benzopyran-4-one [7]. However, its informal name has prevailed, and comes from the Latin word quercetum, which translates as “oak forest” [8]. It can also be found as 3,3′,4′,5,7-pentahydroxyflavone, and its basic chemical structure follows that of the flavonol family [9]. The molecule of quercetin consists of a basic flavonoid skeleton, that is, two benzene rings attached (A, B) to a heterocyclic pyrene (C), and differs from the other flavonoid compounds due to the position of five hydroxyl groups, one at position 3 of ring C, two at positions 3’,4’ of ring B, and another two at positions 5 and 7 of ring A. Quercetin, like other flavonoids, binds to monosaccharides, disaccharides, and sugars with more than three molecules, such as glucose, galactose, rhamnose, xylose, arabinose, and rutinose [10,11]. These bonds are called glycosidic bonds and the most common glycosidic bond produced by quercetin is the O-glucosidic bond, and in particular, the 3-O-glucosidic bond, which is formed between the carbon at position 3 in ring A and its complexes. The 7-O-glucose bond also appears in the quercetin derivatives, whereas the γ-glucose bond, which develops with the 6 carbon of ring A, is rare [12]. Quercetin occurs to a very large extent in the form of glucosides, and in particular, the most common forms are quercetin-3-glucoside and rutin [13,14,15]. An amount of quercetin aglycone has also been found in the saliva where it is prone to oxidation by pro-oxidases during its stay in the oral cavity. The oral cavity also possesses enzymes capable of hydrolyzing quercetin glycosides, but not rutin and quercetin aglycone, as demonstrated by Nemeth and Piskula [16].

The quercetin derivatives mentioned above cannot be absorbed in the form of glycosides, so they must undergo cleavage to remove the hydrocarbon, sulfate, or methyl group from the basic skeleton of the quercetin aglycone form, which can eventually be absorbed [17]. The basic enzymes that take part in the deglycosylation of quercetin are found in the intestinal mucosa and are primarily associated with lactase-phlorizin hydrolase or lactase, which belongs to the group of hydrolases that hydrolyzes disaccharides and compounds with O- or S-glycosidic bonds [18,19]. The epithelial enterocyte membrane also contains sodium-glucose protein transporter 1 (SGLT-1), which carries glycosylated molecules along the outer part of the mucosal enterocyte membrane in the intestinal tract [20,21]. According to Walgren et al. [22], glucose transporter 2 (GLUT-2) and the associated multidrug-resistant protein 2 (MRP2) may also be involved in the absorption process. The absorption of quercetin glycosides has been found to be more efficient than that of quercetin molecules without the glycosyl group, and there is a greater delay in the absorption of rutin, which cannot be deglycosylated in the small intestine so it is transmitted intact to the large intestine to be processed by mucosal microbial enzymes [16,23]. Furthermore, a previous study by Hollman et al. [23] on patients with ileostomy revealed that a significant amount of quercetin is absorbed from the gastrointestinal tract, with glycosides being the most readily absorbed, as reported by several recent studies [17,21,24,25,26,27,28,29]. Glycosides and associated forms of quercetin that are not processed in the small intestine can be degraded by colon-secreting microbial enzymes such as aL-rhamnosidase and β-glucosidase [30]. After the absorption of quercetin, the metabolic pathway occurs in two stages, phase I and II [16,21,25,31,32]. Phase I of the metabolic process is characterized by oxidative reactions and resembles the metabolic phase of apigenin [33,34]. This is followed by phase II, which is more complex and is characterized by the several reactions. Specifically, the reactions of O-methylation, sulfation, and glucuronidation take place in enterocytes and the result is the production of quercetin metabolites, which are released into the bloodstream to follow various metabolic pathways or to take part in catabolic reactions forming phenolic derivatives of lower molecular weight [17,33]. Sulfotransferases function primarily in the liver and their role is the transportation of a sulfate moiety from 3′-phosphoadenosine-5′-phosphosulfate to a hydroxyl group in the quercetin molecule, thus producing quercetin sulfate derivatives [34,35]. The metabolic stage of glucuronidation consists of UDP-glucuronyltransferases (UGT), producing O- and N-glucuronides, and occurs in both hepatocytes and enterocytes [16,36]. “Intestinal recycling” takes place locally in enterocytes after the absorption and biotransformation of quercetin into phase II metabolites with the help of the cytosolic enzyme β-glucosidase, which is located in the membrane of mucosal cells. Quercetin metabolites are therefore subject to repetitive hydrolysis, resulting in the formation of enterocyte-absorbed quercetin aglycone [25]. With the arrival of quercetin metabolites from cleavage in the intestinal tract as well as the stomach and liver, the derivatives that emerge via the portal vein or lymph have undergone a phase II metabolic process (glucuronidation, sulfation, o-methylation) and are subject to oxidative reactions [37]. What is mainly found in the nephrons are transport polypeptides of organic anions (OATPs, OATs) from the family of soluble cell membrane carriers (SLC), and in particular, polypeptides 1 and 3 (OAT1, OAT3), which are responsible for the secretion of phase II metabolites into the urine [38,39].

Approximately 350 different quercetin conjugates have been found in plants, 180 of which were identified in 2001–2003 [12]. Glycoside formation in plants is catalyzed by UDP-dependent glycosyltransferase (UGT), which is thought to be responsible for the attachment of sugars, mainly to the 3′-position of the phenolic ring of the quercetin molecule [40]. During the process of metabolism, new quercetin derivatives are formed that are more stable for circulation in the blood plasma, which result from reactions of glucuronidation, methylation, and sulfation [17,41]. These new complex molecules therefore consist of glucuronic acid, methyl and sulfide groups, quercetin monoglycuronide, quercetin diglucuronide, quercetin sulfate, quercetin sulfate monoglycuronide, and methylated quercetin monoglycuronate [34]. O’Leary et al. [36], located quantities of methylated quercetin glucuronides in HepG2 hepatocytes, with the methyl group located at positions 3′ or 4′ and glucuronic acid at position 7, forming the metabolites 3′-methylquercetin-7-O-glucuronide and 4′-methyl quercetin-7-O-glucuronide. Furthermore, through catabolic reactions carried out by microbial flora (comprising *Pediococcus* spp., *Streptococcus* spp., *Lactobacillus* spp., *Bifidobacterium* spp., and *Bacteroides* spp.) in the colon, stomach, liver, and kidneys, the complex molecules are mainly broken down into phenolic acids, such as 3-hydroxyphenolic acid [17]. Quercetin can be a powerful antioxidant by creating complexes after taking into account its chemical properties related to its high solubility and bioavailability [42,43,44]. Its action against diabetes is explained by its effect on hepatic HepG2 cells, where it alleviates oxidative stress due to hyperglycemia, and activation of the Nrf2 metabolic pathway in pancreatic tissue [45,46]. Moreover, in the clinical field, Rauf et al. [47] stressed that quercetin is both an inhibitory and a preventive agent for cancer, and can assist treatments against the disease, such as chemotherapy.

## 2. Bioactivity

### 2.1. Antioxidant Action

The breakdown of active oxygen radicals can be achieved by hydrogen atom transport (HAT), simple electron transport followed by proton transfer (SET-PT), sequential electron transfer with proton loss (SPLET), and by the chelation of transitional metals (TMC), as shown on Table 1 [48,49,50,51]. In fact, these mechanisms are carried out simultaneously at different rates [51,52,53,54].

More generally, it is argued that the chemical structure plays an important role in the antioxidant capacity of quercetin, since the hydroxyl groups (OH) mainly in the B rings and C rings seem to contribute more to the antioxidant activity of quercetin and glycosides compared to ring A [51]. Quercetin, and in particular orthokinone (QQ), takes part mainly in reactions with the enzyme glutathione (GSH) resulting in the formation of two non-reactive metabolic products, 6-glutathioniyl-quercetin (6-GSQ) and 8-glutathioneyl-quercetin (8-GSQ) [55]. A metabolic derivative of quercetin, 4′-methyl-quercetin (4′MQ), is an important product that contributes to the antioxidant activity of quercetin. According to Moalin et al. [56], who studied the reactivity of thiol in the ascorbate–glutathione cycle and the presence of quercetin and its derivatives, 4′-methyl-quercetin has a corresponding oxidation product, 4′-methyl-quercetin oxidized 4′-methyl-quercetin (ox4′MQ), with thiol reactivity 350 times lower than the fatty metabolic product of aglycone quercetin. Several studies show that quercetin has the ability to reduce cellular oxidation as an antioxidant agent, with one of the mechanisms in which it participates being an increase in the GSH:GSSG quotient while reducing the levels of mixed disulfide proteins [57,58,59]. However, Gao et al. [60] showed on the other hand that the presence of quercetin reduces the amount of glutathione in liver cells, resulting in a small advancement of the oxidative process. In this way they also highlight the other side of quercetin that concerns its pro-oxidative action. Dixon et al. and Hasanuzzaman et al. [61,62] have used the terms “antioxidant recycling” and “antioxidant network” in their research [63] to refer to the interaction of ascorbat e–glutathione cycle with quercetin and its oxidized products.

In the research conducted by Banik and Bhattacharjee [64], the important role of polyphenolic compounds was proposed, including the quercetin molecule, as one of the most promising secondary metabolites. These metabolic products are formed following the biochemical pathways of chalcone synthase and with the contribution of cinnamic acid. The presence of polyphenols in the ascorbate–glutathione cycle (ASC-GSH) achieves better regulation of redox and the reduction of salinity stress during early germination in rice. Bu et al. and Mi et al. [65,66] noted that quercetin supplementation significantly restored the level of glutathione depletion and the activities of the enzymes superoxide dismutase (SOD) and glutathione peroxidase (GSH-Px). On the other hand, Gao et al. [60] confirmed hypotheses from previous studies [55,67,68] by demonstrating that the presence of quercetin reduces glutathione in liver cells by promoting the oxidative process by a small percentage, thus demonstrating the pro-oxidative action of quercetin concerning the production of oxidative substances. Further, quercetin is able to trigger reactive metal ion chelating reactions and use the reduction caused by glutathione in order to restore free radicals to their original state [69]. A study by Tvrdá et al. [70] confirmed the research of Boots et al. [71], who expressed the hypothesis that quercetin suppresses the formation of free radicals at various levels by inhibiting the formation of peroxide root through chelation of iron (reduction of hydroxyl root formation rate) and by inhibiting the formation of lipid peroxide.

### 2.2. Absorption and Bioavailability

The processes carried out for the absorption and final utilization of quercetin significantly reduce its bioavailability [72,73]. Although polyphenols are characterized by low bioavailability (about 20% for quercetin) compared to macronutrients (bioavailability of 90%), data from previous pharmacokinetic studies confirm that flavonols (as a subcategory of polyphenols) have the highest absorption and utilization capacity after phenolic acids and isoflavones [74,75,76]. In addition, studies on the bioavailability of quercetin show that after absorption, more than 96% of is excreted from the body within 72 h [77]. An important step in the absorption and bioavailability of quercetin involves transporters found mainly in the membranes of cells of the oral cavity, stomach, small and large intestine, liver and kidneys. In particular, a protein associated with resistance to multiple drugs (MS2) significantly reduces the bioavailability of the quercetin molecule since it is a transporter that can carry quercetin substrates that come from the intestine back to the lumen of the intestine [36,38]. According to Lee and Mitchell [26], the female sex reacts at a slower rate to the absorption and utilization of quercetin than males, attributing this difference to sex hormones, although without absolute certainty. According to Pressman et al. [78], the absorption and metabolism of bioactive substances in “extreme ages” (i.e., at early and elderly ages) is less active. On the other hand, there are several exogenous factors that determine the bioavailability of quercetin. In particular, the physicochemical structure of the molecule, its molecular weight and its chemical properties, such as solubility, permeability, lipophilicity, hydrophilicity, the ability to create bonds, melting point, and chemical stability, play an important role in its course not only in the human body but also before its ingestion [73,79]. The process of O-alkylation, i.e., the addition of an alkyl group (CvH2v+1) such as methylation with the addition of a methyl group (CH3), helps to improve the bioavailability of quercetin by producing methyl-derivatives [80,81]. Exogenous factors also include the food matrix, i.e., the interaction of quercetin and its derivatives with the nutrients and non-nutrients contained in the ingested food, resulting in the enhancement or inhibition of absorption and availability in the body [74,76]. Due to the interaction of the substances in a food, an ingredient can have a greater effect on the human body than a dietary supplement with the same amount of that ingredient [79].

### 2.3. Cancer

According to recent surveys [82,83], the consumption of foods rich in flavonoids, such as quercetin, reduces the risk of cancer due to a combination of their antioxidant and anti-inflammatory actions. Quercetin inhibits and prevents cancer, but also aids in recovery after treatments against the disease, such as chemotherapy [47]. The anticancer properties of flavonoids, and especially in this case, of quercetin, are attributed to their chemical structure [48]. Quercetin helps in the proper functioning of the mitochondria, affects the phases of the cell cycle and autophagy, and inhibits the proliferation of mesothelioma cancer cells and the progression of metastasis. It contributes to cellular apoptosis, it is a buffering factor in cell signaling pathways, such as Wnt/beta-catenin, PI3K/Akt/mTOR, MAPK/ERK1/2, NF-kB, JAK/STAT, and Notch, and plays an important role in the inhibition of angiogenesis, which is mainly responsible for the occurrence of metastases in different organs [84,85,86]. In addition, quercetin acts as an anticancer agent by inhibiting the action of enzymes suspected of carcinogenesis and binds to proteins and cell receptors [47,87]. Earlier, Ferry et al. performed a phase I clinical trial, investigating the pharmacokinetic effects of intravenous quercetin injection in 51 cancer patients (large bowel, ovary, pancreas, melanoma, stomach, renal, hepatoma, and non-small cell lung) at doses of 60–2000 mg/m^2^. It was found that a dose of 945 mg/m^2^ was safe. Higher doses could cause vomiting, high blood pressure, nephrotoxicity, and decreased serum potassium. The achieved plasma levels inhibited lymphocyte tyrosine kinase activity and showed signs of anticancer activity [88]. Due to the lack of clinical trials regarding the effect of quercetin as an anticancer agent, more research needs to be conducted [89].

Another study investigated the anticancer effect of quercetin in vitro and in vivo using mice and the human colon cancer cell lines, DLD-1 and HT-29. After examining the cells in vitro, they were injected into 4-week-old Balb/C mice to stimulate human colon cancer xenografts. The hypothesis of the study was that quercetin might be able to sensitize the cancer cells by making them vulnerable to radiation, thus improving the treatment. The treatment and control groups were treated with radiation for four weeks (5 Gy/week) and/or 10 mg/kg per day quercetin. They found that a combination of quercetin and radiation showed a benefit in the tumor-bearing mice by reducing the tumor size significantly (*P* < 0.01) compared to the administration of quercetin or radiation alone. The study also revealed a restraint in the expression of Notch-1, Jagged 1, Hes-1, Presenilin 1, and Nicastrin [90]. Moreover, Sundaram and colleagues treated human cervical carcinoma HeLa cells with increasing doses of quercetin from 1 μM up to 150 μM for 1 to 2 days and the results showed that 25 μM and 50 μM decreased the enduringness of the cells by 13% and 20% after 24 and 48 h and 23% and 48% after 24 and 48 h, respectively [84].

Doğan et al. researched the potential protective role of quercetin in vivo on rat fetal brain tissue exposed to chemotherapy prior to pregnancy. A dose of 10 mg/kg per day of quercetin, suspended in corn oil, was orally administered throughout the study, starting 3 days prior to the beginning of chemotherapy, and impregnation occurred 2 days after chemotherapy with cyclophosphamide (CYC) and doxorubicin (DOX) was commenced. The results showed a statistically significant benefit when quercetin was administered with CYC or DOX by decreasing the levels of oxidative factors, such as superoxide dismutase (SOD) and malondialdehyde (MDA), while increasing the availability of glutathione (GSH) and catalase (CAT), which combat cell oxidation. Moreover, quercetin reduced the toxic effects of chemotherapy on the fetus [91]. DOX is an efficient agent for breast cancer chemotherapy, but it is toxic for non-tumor tissues, especially myocardial cells, limiting its application. Quercetin enhances the cytotoxicity of DOX for tumor cells and reduces cardiotoxicity [92].

A recent study showed that quercetin can re-sensitize drug-resistant breast cancer cells MCF7-DR (MCF7 cell line resistant to docetaxel (DTX)). The results showed that quercetin inhibited lymphoid enhancer-binding factor-1 (Lef1) expression in a dose-dependent manner and also decreased the expression of TGF-β, indicating there is a relationship between Lef1 and TGF-β [93]. Alhakamy et al. used a formulation of quercetin optimized with scorpion venom peptides (SV) and phospholipon (PL) against MCF-7 and the results indicated an increase in caspase-9, Bax, Bcl-2, and p53 mRNA expression after treatment as well as a significant reduction in the activity of TNF-α and NF-κB compared to the control formula and quercetin alone. A comparison of IC_50_ values proved that the optimized quercetin formula was more effective against MCF-7 cells than the control formula and quercetin alone [94]. A human metastatic ovarian cancer cell line (PA-1) was used by researchers to examine the antiproliferative activity of quercetin after treatment with different concentration of quercetin (0–200 μM) for 24 and 48 h, identifying dose-dependent inhibition of cell growth with an IC_50_ value of 75 μM for a 24 h treatment. Quercetin inhibited the growth of PA-1 cells by modifying the endogenous apoptotic pathway by upregulating pro-apoptotic Bcl-2 family member gene expression and decreasing anti-apoptotic Bcl-2 family member genes. Furthermore, cytochrome c and caspase-9 and -3 protein expression was also increased [95]. Ovarian cancer SKOV-3 cells were treated with quercetin and it inhibited proliferation in a time- and dose-dependent manner, causing cell cycle arrest in the G0/G1 phase and a significant decrease in the percentage of cells in the G2/M phase. Furthermore, it appeared that quercetin induced SKOV-3 cell apoptosis and protein expression levels in the surviving cells decreased as the concentration of quercetin increased. The results indicate that quercetin inhibited the proliferation of SKOV-3 cells in vitro by inhibiting cell cycle progression and inducing cell apoptosis [96].

Quercetin was used by Kim et al. to sensitize pancreatic cancer cells to TRAIL-induced apoptosis. Their research showed that quercetin down-regulated the levels of cFLIP through JNK-mediated degradation, and thus, cells became sensitive to TRAIL-induced apoptosis. Thus, cFLIP may be an attractive therapeutic target for pancreatic cancer therapy [97]. Quercetin has also been shown to have a synergistic effect on the anti-cancer effect of gefitinib on PA-1 ovarian cancer cells. Thus, the combination of quercetin and gefitinib showed higher cytotoxicity than either compound on its own [98].

### 2.4. Diabetes

Flavonoids, thanks to their antioxidant and anti-inflammatory characteristics, can give the body and the immune system a boost to prevent the onset of diabetes and regulate glucose more effectively. Type 2 diabetes mellitus is the most common type of diabetes, accounting for up to 90% of the total population with insulin resistance and decreased insulin secretion. According to the data so far, treatment is aimed at increasing insulin secretion and lowering blood glucose [99]. Although the antioxidant and anti-inflammatory effects of quercetin in the body are considered to be indisputable, this polyphenol also affects mechanisms related to the rate of insulin secretion and the absorption of glucose by cells [100,101]. In addition, the flavonoids may have an anti-hyperglycemic action and can affect the regulation of hormones and peptides involved in the metabolic pathways of the glucose-insulin system [102,103].

Administration of quercetin in an amount of more than 500 mg/day for more than 8 weeks is capable of bringing about a decrease in blood glucose levels [104]. Shi et al. [103], in their meta-analysis, investigated the effect of quercetin on the glycemic and lipid profile of laboratory animals, citing research based on diabetic rats vaccinated with streptozotocin. Doses of quercetin ranged from 2.5 to 80 mg/kg/day with a duration of administration of 2 to 10 weeks. Lachin [105] observed a decrease in blood glucose and microalbumin in the urine, and an increase creatinine, after administration of 200 mg/day of a sweet cherry extract to Wistar rats for 30 days. Another recent study employed a mixture of anthocyanate, hydrocyanic and flavonolic acid isolated from sweet cherry to show that 25 μg/mL had a synergistic effect on the absorption of glucose by hepG2 liver cells that was mainly due to flavonols and coumaric acid [106]. A combination of 70mg of sitagliptin and 50 mg/kg quercetin improved glycemic control, the metabolic profile, and the pancreatic condition in rats [107]. Research conducted on a Chinese population by Yao et al. [108], showed that 20.9 mg/kg/day (±2.32 mg) quercetin significantly reduced the risk and promoted the healing of metabolic syndrome, as shown in Table 2.

Many studies have been conducted on the combination of quercetin with other substances and their effect on diabetes mellitus. Eitah et al. demonstrated in diabetic rats the effectiveness of a quercetin/sitagliptin combination for achieving adequate glycemic control and proper β-cell integrity and function by significantly improving hyperglycemia, hyperlipidemia, oxidative stress, and inflammatory load on β-cells. The results of the combination were better than either compound alone, so this combination was proposed as an effective approach in the fight against diabetes [107]. Furthermore, Abdelmoaty et al. injected quercetin (15 mg/kg/day) into rats before streptozotocin (STZ) administration and continued injecting quercetin for 25 days. Quercetin was able to prevent STZ-induced diabetes and reversed the inhibitory effect of STZ on the activity of antioxidant enzymes such as glutathione peroxidase (GSHPx), superoxide dismutase (SOD), and catalase (CAT) in the pancreas [109]. Another study in rats fed a high-fat high-sucrose diet has shown that the combination of a glycosylated quercetin derivative and soyabean fiber prevented dietary glucose intolerance and was accompanied by an increase in plasma GLP-1 levels. In addition, GLP-1 levels were positively correlated with quercetin concentrations in plasma, thereby demonstrating the effect of soybean fiber and quercetin on reducing the risk of glucose intolerance and imbedding diabetes [45].

There have been some promising clinical studies for the use of quercetin as a potential drug for diabetes and its complications. Mazloom et al. performed a clinical study on 47 patients with type 2 diabetes and reported that quercetin supplementation (250 mg/day) for 8 weeks had no significant impact on glycemic control. However, the results showed that quercetin supplementation significantly improved total antioxidant capacity and significantly reduced atherogenic ox-LDL levels in serum [110]. Hickson et al. studied patients with diabetic kidney disease after oral administration of a combination of dasatinib (100 mg) and quercetin (1000 mg) for three days. The combination reduced adipose tissue senescent cell burden along with many other important factors, including IL-6, IL-1a, fibroblast growth factor (FGF-2), and MMP-9 [111]. In another clinical study, Galleli et al. tested the effects of hyaluronic acid or a nano-hydrogel embedded with quercetin and oleic acid in 56 diabetic (DM) patients with diabetic foot ulcers that had not responded to mechanical compression. The nano-hydrogel treatment significantly reduced wound healing time compared to hyaluronic acid without side effects [112].

A recent study fabricated an quercetin nanoemulsion that showed good stability for 45 days and had higher oral bioavailability and release compared to pure quercetin. Diabetic rats administered 12.5 mg/kg quercetin showed significant protective and therapeutic antidiabetic effects against STZ-induced diabetes by controlling body weight and blood glucose levels and reducing serum lipid levels. Furthermore, quercetin significantly inhibited tissue damage and oxidative stress indicators [113]. Another nanoemulsion containing quercetin recently reported enhanced bioavailability and an antidiabetic effect. A liquid self-nanoemulsifying drug delivery system (L-SNEDDS) for curcumin and quercetin was prepared and solidified using Ganoderma lucidum extract and probiotics and further converted into pellets. This system provided a great increase in the bioavailability of curcumin and quercetin as well as good stability, and was able to restore normal blood levels of glucose, lipid antioxidant biomarkers, and pancreatic and liver tissue architecture in STZ-induced diabetic rats without side effects [114]. In another study with diabetic rats, quercetin reduced hepatotoxicity, a complication associated with diabetes [115]. Dong et al. investigated the effect of quercetin in type 1 diabetic rats and their findings after a 4-month treatment indicated that quercetin ameliorated oxidative stress-induced cell apoptosis of seminal vesicles via the activation of Nrf2 [116].

Diabetic nephropathy (DN) is a common microvascular complication of DM that may lead to end-stage renal disease (ESRD). Quercetin was shown to protect the kidney and delay renal intestinal fibrosis while ameliorating many biochemical parameters [117]. In a recent study on another complication of diabetes mellitus, diabetic cardiomyopathy, diabetic rats were treated with quercetin and its effects on heart tissue were assessed. Diabetes accelerates the formation of reactive oxygen species (ROS) that are involved in the pathogenesis of heart failure, so an antioxidant molecule such as quercetin may protect the heart and delay the damage. The results shown that quercetin exhibited a therapeutic effect on diabetic cardiomyopathy [118]. Ojo et al. reported that administration of both quercetin and vitamin E ameliorated cardio-apoptotic risk via inhibition of mPT pore and the regulation of mitochondrial apoptosis [119].

### 2.5. Dietary Concentration, Supplements, and Toxicity

A variety of foods that can be considered quite popular throughout the population have a content of quercetin over 25 mg/100 g. According to Table 3 and the USDA database of flavonoid concentration in foods [120], the highest content of quercetin is contained in *Capparis spinosa*, either in its raw form (233.84 mg/100 g) or packaged (172.55 mg/100 g). Other widely used foods with a high concentration of quercetin include raw radish leaves (70.37 mg/100 g), raw wild arugula (*Diplotaxis tenuifolia*) (66.19 mg/100 g), raw radishio (*Cichorium intybus*) (31.51 mg/100 g), fresh dill (*Anethum graveolens*) (55.15 mg/100 g), raw coriander leaves (52.90 mg/100 g), raw fennel leaves (48.80 mg/100 g), and fresh oregano originating in Mexico (42.00 mg/100 g). A significant amount of quercetin is also contained in the yellow raw hot pepper (50.63 mg/100 g) as well as the ancho variety (27.60 mg/100 g) and red spring onion (30.60–39.21 mg/100 g).

Quercetin is found in nature in different forms either as a composite molecule bound to additional molecules, such as sugars, sulfates and methyl groups, or in its simple form. According to the above studies, however, by recording the frequency and quantity of quercetin in a typical diet, it seems that quercetin intake is low, either due to a diet poor in organic products or the consumption of foods with low in quercetin [120,121,122,123,124,125,126]. The distribution of quercetin content in a wide range of foods occurs mainly from little to moderate concentrations. It therefore presents a degree of difficulty for the consumer to obtain a high amount of flavonol daily. In this regard, quercetin supplements are an important means of enhancing daily intake. They are mainly used for their antioxidant and anti-inflammatory actions according to the instructions of an attending physician, pharmacist, or on the recommendation of a nutritionist.

Finally, some foods less widely used in European cuisine are ranked high based on the concentration of polyphenols, suggesting that they are a good source of antioxidants. In particular, 100 g of hartwort leaves contain 29.30 mg of quercetin; raw watercress (*Nasturtium officinale*) has a concentration of 29.99 mg/100g; carob flour has a concentration 38.78 mg/100 g; green unripe and ripe juniper berries have a content of 42.81 mg/100 g and 46.61 mg/100 g, respectively; concentrated aronia juice contains 68.17 mg/100 mL; and concentrated sambucu juice has a quercetin concentration of 108.16 mg/100 mL.

In addition, 200–1200 mg of quercetin daily can be administered as a dietary supplement with no signs of toxicity [127,127,128,129,130,131]. In particular, Lee and Mitchell [26], in a study conducted on the absorption of quercetin from apple and onion, showed that a fatty meal or the presence of apple pectin, oligosaccharides, and lecithin, increased the absorption and effectiveness of quercetin and its glycosides. Previously, it was widely believed that quercetin was a mutagenizing agent with a high risk for the appearance of carcinogenic cells, which has limited its use in food enrichment [130]. At this point, it is worth noting that according to the monographs of the IARC on the identification of carcinogenic risks to humans, quercetin has ceased to be considered a carcinogenic substance [132]. In addition, according to Okamoto [133], quercetin does not cause toxicity and is not likely to be a mutation and carcinogenicity factor when administered in the typical frameworks due to the low bioavailability of flavonols after introduction into the body. Only after administration of extremely large doses is a high concentration of free aglycone quercetin in the body considered possible; however, the body has mechanisms for the rapid elimination of “foreign” substances, and quercetin remains only for a short period of time. In addition, a study conducted on patients with chronic obstructive pulmonary disease administered quercetin in doses of 500 mg/day, 1000 mg/day, and 2000 mg/day for one week, and no toxic effects were observed for any dose [134].

## 3. Discussion

Quercetin follows a metabolic pathway similar to that of drugs and foreign substances and polyphenols are characterized by low bioavailability. A meta-analysis by Di Lorenzo et al. [74]. observed that the consumption of fatty meals improved the absorption and bioavailability of flavonols. Other studies have shown that glycosylated derivatives of quercetin have a better antioxidant effect and bioavailability.

Several studies have investigated the beneficial effects of quercetin on various cancers. The results of in vitro and in vivo studies have been encouraging, demonstrating both the chemoprotective ability of quercetin and its anti-cancer activity that targeted cancer cells without affecting normal cells. By investigating combinations of quercetin with other anticancer drugs (e.g., gefitinib) or methods such as radiation, quercetin has been shown to work synergistically compared to drug or treatment alone. The combination of quercetin and radiation is a promising anticancer therapy due to its low cytotoxicity for healthy cells and its effect on target cells through the regulation of signaling pathways. The targeted effect of quercetin on cancer cells has been supported by other studies such as by Sundaram et al. [84] that proved lymphocytes were not affected by treatment, and therefore, quercetin does not adversely affect normal cells. Quercetin also appears to have a chemoprotective effect, since its administration before chemotherapy reduced the toxic effect of chemotherapy and increased the antioxidant status, so the researchers recommended the administration of quercetin to women who are about to become pregnant after treatment.

Findings on the anti-cancer activity of quercetin are mainly derived from vitro and in vivo studies. The published results of clinical trials are few, such as Ferry et al. [88], but they also indicate quercetin has anti-cancer activity.

Much research has also been conducted on the effect of quercetin on diabetes. Although in vivo studies in diabetic rats have shown a beneficial effect on glycemic control, clinical trials have shown no such effect. However, both in vivo studies and clinical trials have shown the protective effect of quercetin on diabetes-related complications, such as oxidative stress, hepatotoxicity, diabetic nephropathy, tissue damage, diabetic kidney, diabetic foot ulcer, and cardiomyopathy.

Research has shown, as in cancer, that the combination of quercetin with other substances such as sitagliptin and soybean fiber has better antidiabetic effects than either substance alone. Another problem that scientists have been concerned about is the low bioavailability of quercetin, so they have been looking for solutions to increase its bioavailability. Indeed, it was found that administration of quercetin in the form of a nano-emulsion or nano-hydrogel resulted in better bioavailability and release. The findings so far suggest that quercetin shows anti-diabetic activity, possibly indicating this phytochemical drug may be an alternative for managing this disorder and its related complications without side effects.

The beneficial effect of quercetin on cancer and diabetes may be related to its antioxidant activity and its effect on the body′s antioxidant capacity. In both diseases, an increase in oxidative stress levels is observed along with a decrease in antioxidant enzymes. Thus, further study of quercetin and its effects may help us understand the role of oxidative stress and antioxidants in both disease processes.

## 4. Conclusions

Quercetin is a molecule that seems to act as a protective agent for many diseases such as cancer and diabetes and is used in many therapeutic schedules to treat them. Nevertheless, further research is needed to determine an effective and safe recommended daily dose of quercetin that is beneficial to the body and prevents disease.

## Figures and Tables

**Table 1:** Antioxidant mechanisms HAT, SET-PT, SPLET.

Name	Chemical Reaction
SPLET	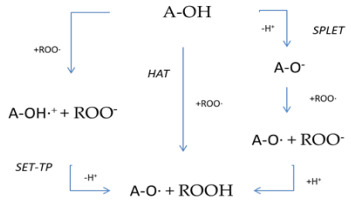
HAΤ	
SET-PT	
TMC	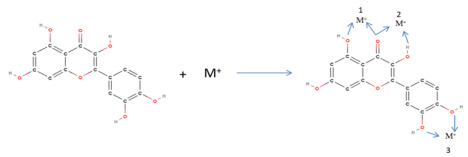

**Table 2:** Research and meta-analysis of the effect of quercetin on diabetes mellitus.

Type	Method	Results	References
Research	>500 mg quercetin/day for >8 weeks	Lowering of blood glucose	[104]
Meta-analysis	2.5–80 mg quercetin/kg/day for 2–10 weeks in diabetic rats vaccinated with streptozotocin	Protection against oxidative damage of beta-pancreatic cells by inhibiting the action of α-amylase and α-glucosidase	[103]
Research	70 mg sitagliptin + 50 mg quercetin/kg	Improvement of glycemic control, metabolic profile + oxidative status	[107]
Research	200 mg quercetin/day of sweet cherry for 30 days in Wistar rats	Lowering of blood glucose + microalbumin in the urine and reduction of creatinine	[105]
Research	20.9 mg ± 2.32 mg quercetin/kg/day Chinese population with DMII from a nutritional source	Reduction of the risk and improvement of DMII	[108]
Research	A sweet cherry phenolic-rich extract (PRE) with concentrations of 0.2, 1, 5, and 25 μg/mL, with 25 mmol/L D-glucose	Promoted HepG2 glucose consumption by 22.8%, 22.9%, 31.8% and 38.6%	[106]

**Table 3:** Foods rich in quercetin based on the USDA Database of Flavonoids in Selected Foods.

	Food	Average Content (mg/100 g)
1	Pepper, ancho	27.60
2	Cauliflower, leaves	29.30
3	Watercress, raw (*Nasturtium officinale*)	29.99
4	Onion, fresh, red, bulb	30.60
5	Radish, raw (*Cichorium intybus*)	31.51
6	Carob, flour (*Ceratonia siliqua*)	38.78
7	Onion, red, raw	39.21
8	Oregano, Mexican, fresh	42.00
9	Juniper berry, green, unripe (*Juniperus communis*)	42.81
10	Juniper berry, ripe (*Juniperus communis*)	46.61
11	Fennel, leaves, raw	48.80
12	Pepper, hot, yellow, raw	50.63
13	Coriander, leaves, raw	52.90
14	Dill, fresh (*Anethum graveolens*)	55.15
15	Rocket, wild, raw (*Diplotaxis tenuifolia*)	66.19
16	Concentrated juice, aronia	68.17
17	Radish, leaves, raw	70.37
18	Concentrated juice, elderberry	108.16
19	Capers, canned (*Capparis spinosa*)	172.55
20	Capers, raw	233.84

## Data Availability

Not applicable.
